# Poisoning by Coal Gas of Two Comanche Chiefs

**Published:** 1886-02

**Authors:** H. W. Moore

**Affiliations:** Fort Worth, Texas


DANIEL'S

### Medical ★ Journal,

PUBLISHED MONTHLY AT

AUSTIN, TEXAS.

Vol. I.] FEBRUARY, 1886. [No. 8.

*"Scribimus indocti, doctique!"*

### Original Articles.

☞ Contributed Exclusively to this Journal. ☜

*The Articles in this Department are accepted, and published with the understanding that we are not responsible for, nor do we indorse the views and opinions of the writers, by so doing.*

### POISONING BY COAL-GAS, OF TWO COMANCHE CHIEFS.

*By H. W. Moore, M. D., Fort Worth, Texas.*

For Daniel's Texas Medical Journal.

IT is so seldom that we come in contact with incidents of poisoning by coal-gas, that, actuated by a disposition to add my mite to the literature of our profession, I have concluded to report the following case, occurring in the noted characters of Quanah Parker and Yellow Bear, both holding the position of chief in the Quohado branch of the Comanche Indians, now resident in the Indian Territory.

On the evening of the 21st of December, 1885, the Indians referred to registered at the Pick-Wick Hotel, in this city. After taking in the town, the usual custom of that class of visitors, they returned to the hotel about midnight. Their tour through the town was characterised by more peace and quiet than their invasions into civilized communities but a few years ago, or than would be phrenologically indicated by the well developed bump of combativeness peculiar to the men of that rapidly disappearing race.

Yellow Bear, going to his room first, turned out the light and retired to rest, little dreaming that this sleep was to be his last —that the angel of death was even then hovering over him, ready to waft his soul to the realm of immortality. In a short time his companion followed him to the room, and relit the gas, preparatory to going to bed. Just before lying down, he turned out the light, but in so doing must have unconsciously re-turned the stop-cock, thus permitting the slow escape of gas. This is the only theory by which we can account for the accident, since he claims to understand the management of this variety of artificial light.

Judging from the time they were exposed to this vitiated atmosphere, the escape of gas must have been very slow, and not equal to the full capacity of the burner, for the room occupied was very small—the door and transom both closed—which, with the window down, rendered the ventilation very inferior; and in my opinion life would have been extinct in both, several hours before the discovery of the situation, had the gas been escaping in volume equal to the full capacity of the burner.

As near as we can determine, it was about one o'clock a. m. when Quanah Parker retired, and this hour marks the commencing gaseous impregnation of the atmosphere in the room. Thus, in calculating from that time until 1:30 p. m. of the following day, these persons were exposed to the toxic action of this agent, for a period of more than twelve hours. Whereas, it has been proven by experiment that animals thrown into a room where gaseous carbon has been evolved to a point of saturation, a termination of life is reached by the victim of experiment in two or three minutes. Consequently we are justified in presuming that, notwithstanding the deficient ventilation, the escape of carbon from, and the entrance of oxygen into the room, was accomplished to a limited extent, at least; and that the accumulation of gas was gradual and slow, though the supply was, to the limited area within the room, greatly in excess of oxygen, and furnished much more rapidly than it was possible for its elimination through the channels of exit offered by the crevices formed in connection with the window, door and transom.

Resuming the thread of our report, Quanah Parker stated, when convalescing, that he detected, soon after going to bed, the odor of escaping gas, but could not account for its presence, and, unfamiliar with the nature of the danger to himself and companion resident in this toxic agent, he drew the cover over his head to shut out the offensive odor, and was soon soundly sleeping, to be awakened later (how much later it was impossible for him to form even an estimate). He was aroused by a burning, parched sensation of the lips, mouth and fauces; a horrible constriction throughout the air passages, pain in the chest, extending down the arms to the elbow; a feeling as if there were heavy weights bearing down upon the thorax; and all the frightful symptoms of a dangerous asphyxia.

The venous system was already charged with carbonized blood, and the arteries and arterioles were gradually filling with it. He aroused his companion, mentioned his symptoms, but to learn of a similar condition there. He left his bed, but, being unable to maintain the erect position, in consequence of cerebral disturbances, he fell to the floor several times in his attempts to walk, producing contusion of the scalp. Nature gradually growing weaker and weaker, under the baneful effects of failure to decarbonize her life-maintaining fluid, was yielding inch by inch to the invading foe, until Quanah, unconscious lay prostrate on the floor.

In this unconscious or comatose condition, he remained until the situation was discovered by the chambermaid, who immediately informed the clerk and manager of the hotel. In response to a summons from them, I hastened to the room occupied by the victims of this unfortunate accident. Entering the room a little before 2 o'clock (this being the evening of the 22d of December), the following situation presented itself: Yellow Bear was lying on the floor, face downward, with mouth and nostrils pressed so firmly against the floor as to interfere very materially with the function of respiration, diminishing very much the supply of air to the lung, and greatly limiting the decarbonization of the blood. A sero-sanguinolent discharge was trickling from the nose, probably the result of capilary rupture dependent upon a fall. Judging from the well developed rigor-mortis and other indications, I reached the conclusion that he had been dead several hours. I can only account for the death of Yellow Bear and the recovery of his companion by the difference in age, he being nearly sixty, which, with its corresponding loss of vitality dependent upon senile decay, rendered him much less able to resist the combined influences on the system of coal-gas, the toxic accumulation of mon-oxide of carbon and the inefficient supply of oxygen so necessary for the decarbonization of the blood. Then, too, the position of Yellow Bear on the floor was, I think, a prime factor in the rapid production of his death, owing to the obstruction it offered to the free ingress and egress of air to the lung. On the other hand, Quanah Parker was a young and vigorous man. He was lying on the floor between Yellow Bear and the window, in the dorsal decubitus, with appearances strongly indicative of rapidly approaching dissolution. There was a white, frothy saliva oozing from his mouth in large quantities; his eyes were fixed in the orbit, and insensible to the touch of the finger when passed over the conjunctiva; the pupils slightly contracted; jaws firmly clenched, so much so that the application of considerable force failed to separate them; respiration irregular and intermittent; marked cyanosis, more especially during the intermission; heart's action very irregular and feeble, first sound absent; no radial pulse, and carotid pulsation very weak; capilary circulation very bad; surface of body and extremities cold, and the skin was bathed in a clammy perspiration.

After opening the door and window, with a view to producing as thorough ventilation as possible, I had the lower extremities elevated to a plane considerably above his head, wrapped him well up in blankets, placed bottles of hot water and hot bricks to the body and extremities, and a mustard plaster to the full length of the spine. Artificial respiration was commenced and continued until, with the assistance of stimulants, there was marked improvement in the respiration and circulation.

Very soon after first seeing him, I gave him a hypodermic injection of fl. ext. belladonna gtt iij, fl. ext. digitalis gtt iv, fl. nux vomica gtt vj. I used the fl. ext. belladonna and fl. ext. nux vomica to avoid the delay incident to procuring a solution of atropia and strychnia (a much more elegant preparation for subcutaneous injection). I next administered nitrite of amyl by inhalation, and continued it until its physiological effect was noticeable.

The hypodermic injections were continued at intervals of thirty minutes or longer, in gradually decreasing doses until reaction was thoroughly established. The nitrite of amyl inhalations were repeated as often as indicated by evidences of heart failure. The respiratory and cardiac functions were gradually restored, after hours of constant watching and careful stimulation; though it was not until after the second hypodermic injection that we could detect the slightest pulsation of the radial artery; and for ten or twelve hours the heart's action continued very weak, failing, through a period of nearly twenty-four hours, to approximate in regularity, volume and strength, a normal condition. It was not until in the early part of the night that I discovered a returning ability to swallow, after which I discontinued the use of the hypodermic syringe, and relied upon the stomach to aid me in the introduction of remedies to the system.

I gave him every two hours through the night, combined with the belladonna, digitalis and nux vomica, carbonate of ammonia in ten grain doses, suspended in a vehicle of mucilage acacia. This mixture was well retained, notwithstanding its frequent tendency to produce gastric irritation.

With the return of consciousness and the ability to articulate, he complained of tightness and pain in the chest, and pain extending down the arm to the elbow, and also pain and fullness above the pubis. Making an examination of the region above the pubis, I found the bladder very much distended. Introducing a Nelaton's soft rubber catheter, nearly two pints of urine were passed, normal in color, odor, etc. The sense of comfort following this operation was very gratifying to the patient. Brandy and water were ordered given freely through the night, but very little was taken in consequence of his aversion to stimulants, very much to my surprise, since the Indians, as a rule, are very fond of all kinds of stimulants, so much so as to constitute a weakness of their race.

December 23. Patient very much improved—his condition under the circumstances, in every way satisfactory. His bowels not having moved since the accident, an enema of warm water and castile soap was ordered, and followed soon after its administration by copious dejections from the lower bowels. Kidneys were acting freely—urine passed without the use of the catheter. Some light nourishment was taken during the morning. The administration of the ammonia carb. mixture was ordered continued through the day at intervals of four hours. The evening examination indicated continued improvement, with the exception of slight rise of temperature (101½) and very troublesome cough. Respiration, 28; pulse, 110. Four grains of Sulph. Quinia and one drop of tr. aconite root was ordered given every three hours. The following mixture for cough:

℞ Ammonia Hydrochlorate.............℈ij

Syr. Senega..........................ʒiij

Morphia Sulph ............ ........gr.j

Syr. Tolu...........................ʒiv

Aqua. Anise.........................ʒiv

Aqua. Elix. Auranti Floris *ad* ........℥iij—M. Sig.

Two teaspoonfuls every three or four hours.

Some might object to the morphia in this case, owing to the depressing effect on the respiratory nerve centres, but the quantity prescribed is so small, the cough so incessant, and patient so much improved, that the ill effects, so slight in their nature from the quantity used, could well be disregarded, when we consider the injury which might arise from a continuance of the cough.

As a counter irritant the following was ordered applied to chest with considerable friction every six hours.

℞ Ol. Sinipis .......................gttxv

Ol. Terebinth .. .................ʒij

Ol. Olivae *ad*.....:.................ʒiv

The directions were systematically followed during the night.

December 24. Remarkable improvement in the condition of the patient. He expressed himself as well in every respect.

Stated that he had enjoyed a splendid night's rest, only disturbed by nurse when time to give medicine. Temperature, respiration and pulse normal, but still annoyed by cough. Auscultation and percussion yielded negative results, or, at least, but little pathological change. In fact, his condition was so much improved, although I advised him to remain here a short time longer, he could not be persuaded to do so; and departed that morning for his home in the Indian Territory. I have been informed since by parties from there that he has completely recovered from the accident, and is in fine physical condition.

The report of this case is made, hoping that it may not prove altogether uninteresting to the profession, since the report, as well as the occurrence, of such tragic accidents are somewhat rare, at least, so far as my observation of the literature on the subject extends. The successful results of treatment add to the interest of the report, in consequence of the almost hopeless condition of the patient when first seen.

Nothing original is claimed in the treatment, unless it be in the use of the ammo. carbonate, which I have never noticed recommended. The idea of its administration was conceived in the knowledge of its magnificent physiological action as a diffusible stimulant, and the well known and demonstrable fact that subsequent to the entrance of the drug into the circulation, it undergoes chemical decomposition, and liberates oxygen in a free state; which, in consequence of its affinity for, it unites with carbon, forming the mon-oxide, which, in turn, upon reaching the capillaries of the lungs, is attacked by inhaled oxygen and converted into carbon dioxide, in which state it is eliminated from the body--an important step in the decarbonization of the blood—thus furnishing us a valuable agent in the treatment of poisoning by coal-gas.

This gas, in its pure state, is a compound, composed of hydrogen carbide, but generally contains more or less nitrogen, moisture, etc.

The effect on the system is much the same under any other circumstances or conditions where we have hyper-carbonization of the tissues and blood, which, if from any cause there is failure to eliminate the *materies-morbi* from the circulation, the venous system is first surcharged with carbon, then the arteries are filled, during which the most alarming symptoms, such as are described in this paper, present themselves; and if not promptly relieved, soon produce death.

Dr. E. J. Beall was present, and assisted me in the management of this case.

Respectfully submitting this paper for the consideration of the profession, it is with the hope that, in its details, it will not prove tiresome to those who, in their leisure, may peruse its pages.

